# Barriers and facilitators to attending and being physically active during recreation time among women incarcerated

**DOI:** 10.1186/s12905-022-01831-w

**Published:** 2022-06-17

**Authors:** Ricky Camplain, Heather J. Williamson, Travis A. Pinn, Sara Shuman, Bethany M. Robinson, Maribeth Evans, Crystal Luna

**Affiliations:** 1 Center for Health Equity Research, Northern Arizona University, 1395 S. Knoles Drive, ARD Building, Suite 140, PO Box 4065, Flagstaff, AZ 86011-4065 USA; 2 Department of Health Sciences, Northern Arizona University, Flagstaff, AZ USA; 3 Department of Occupational Therapy, Northern Arizona University, Flagstaff, AZ USA; 4 Department of Biological Sciences, Northern Arizona University, Flagstaff, AZ USA; 5 Department of Psychology, Northern Arizona University, Flagstaff, AZ USA; 6 Coconino County Sheriff’s Office, Flagstaff, AZ USA

**Keywords:** Criminal justice and health, Physical activity, Questionnaire, Jail, Incarceration, Women’s health

## Abstract

**Background:**

Most women incarcerated in jail are not physically active and do not attend recreation time (rec-time), a time dedicated to being physically active, outside. The purpose of this study was to determine barriers and facilitators to attending and being physically active during rec-time among women incarcerated in jail.

**Methods:**

We recruited and distributed a cross-sectional questionnaire to 100 women incarcerated at the Coconino County Detention Facility (CCDF) in Flagstaff, Arizona from March to July 2020. Women were asked about their experience with rec-time at CCDF, including if they had ever attended, how often they attended, if they exercised at rec-time, activities they participated in, and facilitators, barriers, and benefits to attend rec-time.

**Results:**

Among 99 women who completed the questionnaire, 89% had ever attended rec-time. Most women identified environmental- and health-related facilitators to attending rec-time including enjoying natural light (74%), getting fresh air (83%), a change in environment (62%), and to move around and exercise (72%). Many women indicated environmental-, equipment-, clothing, and motivation-related barriers to attending rec-time. Specifically, women believed there was a lack of equipment (56%) and limited access to proper footwear (49%).

**Conclusions:**

As health and environment are important facilitators and barriers to being physically active among women incarcerated in jail, it is important to identify appropriate environmental and policy interventions to increase the use of rec-time and physical activity. If a correctional facility does not offer rec-time or a similar alternative, one should be established, accessible, and welcoming.

**Supplementary Information:**

The online version contains supplementary material available at 10.1186/s12905-022-01831-w.

## Background

Of the more than 20 million adult Americans that have been or are currently incarcerated, more men are incarcerated compared to women. However, the rate of growth for women imprisonment has outpaced men by more than double with a 700% increase since 1980 [1–5]. Although there has been a substantial increase in incarceration among women, because most people incarcerated are men, women’s health has been overlooked.

Incarceration may contribute to poor long- and short-term health among women. Women tend to gain weight while incarcerated [6]. Almost half of women incarcerated at CCDF reported fair or poor general health, an indicator for subsequent mortality other poor health outcomes [7–9]. A high proportion of women also reported anxiety (56%), depression (48%), and hypertension (18%); and fair, poor, or very poor sleep quality (81%) while incarcerated [10].

The benefits of physical activity (PA) are well established and can be immediate. PA has been shown to help with weight control, offset anxiety and depression symptoms, and improve sleep [11]. Those engaged in regular PA can maintain weight and exhibit improved mental health outcomes such lower rates of depression, anxiety, anger, and stress compared to those who are physically inactive [12–14]. A single bout of moderate-to-vigorous physical activity improves anxiety and depression symptoms [11, 15], decreases blood pressure [11, 16], and improves sleep [11, 17] on the day it is performed. Benefits improve when an individual engages in regular physical activity [11]. Other benefits, such as substance abuse treatment success [18], having a sense of control and achievement, and stress reduction may be of particular importance to individuals incarcerated [19].

Despite benefits of physical activity, physical inactivity is commonly experienced by incarcerated individuals [20]. Women are less likely be sufficiently active compared with the general population of women of a similar age [6]. Among women incarcerated at CCDF, less than ¾ attend time dedicated for outdoor recreational physical activity (rec-time), even when they had permission [21, 22]. Additionally, of women who do attend rec-time, 58% were sedentary and only rarely engaged in vigorous physical activity such as running, push-ups, or sit-ups [22]. Promoting physical activity in jails is an important and conceivably far-reaching investment in the health of individuals incarcerated as physical activity is a holistic approach to improving physical and mental health and potentially reducing medical costs within the criminal justice system. However, little progress has been made in developing appropriate physical activity programming in jails.

The little formative research regarding physical activity within prisons [23, 24] suggest that women incarcerated in prison do not engage in physical activity due to motivation-related reasons [23, 24]. Similarly, 75% of individuals incarcerated in CCDF indicated they were too tired or did not feel like going out to rec-time. However, specific reasons and motivations for being physically active among women while incarcerated in jail have been largely left out of the literature as most studies conducted among incarcerated women are in the prison setting. It is important to understand the differences in physical activity between women incarcerated in jail and prison as prisons are typically run by state or federal governments and are longer-term facilities while jails are typically run by county or city governments and are considered short-term facilities in which most are in pretrial detention and have not been convicted of a crime or sentenced [25, 26]. Additionally, much of the research into the connection between health and incarceration of women either focuses on prison populations or combines prison and jail populations, not distinguishing between the two. Compared to individuals in prison, individuals in jail have higher rates of depression, life dissatisfaction, recent illicit drug use, and mortality for people under 44 years old [27, 28]. Thus, there is a need for more jail-specific data on the connection between health and incarceration among women. The purpose of this study was to determine barriers and facilitators to attending and being physically active during recreation time (rec-time) among women incarcerated in jail.

## Methods

### Study setting and population

We conducted our current study at the CCDF, a regional jail in Flagstaff, Arizona. Individuals incarcerated at CCDF housed in women’s dorms were recruited by a CCDF staff member between March and July 2020 during the COVID-19 pandemic. The study’s focus was rec-time, a time dedicated to being physically active, outside. CCDF allows individuals incarcerated the option of 60 min a day, five days a week, for rec-time, weather and safety permitting. Rec-time has been offered during the COVID-19 pandemic to individuals after a 14-day quarantine period. Each of the four housing units have their own recreation area (space dedicated for rec-time). Each has a concrete floor and is equipped with a bolted down piece of exercise equipment [29]. Women were eligible to participate in the study if they were 18 years or older, had the ability to understand and read English, and were residing in a women’s dorm. We excluded individuals in administrative confinement and severe mental illness dorms. We additionally excluded one participant who returned a blank questionnaire. All aspects of the study were approved by Northern Arizona University’s Institutional Review Board.

### Study design

We conducted a cross-sectional sectional study among women incarcerated at CCDF. Through an iterative process based on direct observation of recreation time, meetings with a consultant who had been previously incarcerated, conversations with women incarcerated, and reviews by a survey expert, we developed a questionnaire to determine barriers and facilitators to attending and being active during recreation time (see Additional file 1). Because CCDF was closed to outside programs, including research, a CCDF staff member played a pre-recorded instructional video on an iPad to the women. Female study investigators explained the purpose of the study as well as the consent document on the video. The staff person reiterated to the women that participation was voluntary and will have no effect on their relationship with the jail, parole hearings, or time served. If women were interested in participating, the staff person distributed an unsealed manila folder with the questionnaire inside. Women had one hour to complete the questionnaires after which the staff person returned to the dorm and collected completed questionnaires sealed in manila envelopes for privacy. Finally, an allowable incentive for participating was distributed to all women who participated.

The staff person recorded the number of women in each dorm, those who watched the video, and those who turned in the sealed envelope and received an incentive to document anonymous participation rates. Of the women incarcerated at the time of the study, 100% of women were recruited, consented, and participated in the study.

### Variables

Women were asked about their experience with rec-time at CCDF, including if they had ever attended, how often they attended, if they exercise at rec-time, activities they participate in, facilitators to attending, barriers to attending, and benefits of attending rec-time. Women were additionally asked about exercising in their living space, their experience with the COVID-19 pandemic while incarcerated, their health, and general information, including demographics.

Participants were asked what day they were booked into CCDF, if they had been previously incarcerated, and their age, race, ethnicity, education, and income level prior to incarceration.

Participants were asked about their general health (excellent, very good, good, fair, poor). Additionally, they were asked if a doctor or other health professional ever told them they had asthma, hypertension, high cholesterol, diabetes, prediabetes, anxiety, depression, bipolar disorder, schizophrenia, post-traumatic stress disorder (PTSD), or attention deficit hyperactivity disorder (ADHD). Participants were also asked to self-report their height and weight. Finally, participants were asked how active they were in their day-to-day life before their current incarceration (very active, somewhat active, somewhat inactive, or very inactive).

Participants were asked if they had ever attended rec-time, how often they attended rec-time, and how often they exercised at rec-time each week. Participants were also asked to check all that apply from a prespecified list regarding what motivates them or would motivate them to attend rec-time, barriers to attending rec-time, what activities they enjoy doing at rec-time, and personal benefits from attending rec-time. Prespecified lists were developed through prior research [30], direct observation of recreation time [31], consults with a formerly incarcerated consultant, and discussions with women incarcerated. Facilitators, barriers, and benefits were not mutually exclusive as participants chose all that applied. All facilitators, barriers, and benefits were qualitatively classified into broader categories by the research team and consultation with a formerly incarcerated consultant. Although facilitators, barriers, and benefits could fit into more than one category, we chose the most appropriate for each item. Following the prespecified lists of facilitators, barriers, and benefits, participants were provided space to describe “other” facilitators, barriers, and benefits with open-ended responses. Finally, participants were asked to determine their top three facilitators to attending and benefits from attending rec-time.

### Data analysis

Descriptive statistics were used to characterize the sample, describe the health of the sample, and determine facilitators, barriers, and benefits from attending rec-time. Open-ended responses were analyzed using a deductive qualitative analysis approach. Predetermined thematic areas based on the questions posed to respondents were used, then summarized.

Analyses were conducted using SAS version 9.4 (SAS Institute Inc., Cary, NC).

## Results

### Demographic and health characteristics

Of the 99 participants, the majority were 44 years old or younger (82.8%), American Indian/Alaska Native (57.6%), had not attended any college (high school diploma, GED, or did not graduate high school) (62.6%), had an annual household income of less than $10,000 (52.5%), and had been previously incarcerated (62.6%, Table 1). The median length of current incarceration was 22 days.

**Table 1:** Demographic and Health Characteristics of Women Incarcerated at Coconino County Detention Facility, 2021 (n = 99)

Characteristic	N	%
*Age*		
18–24	11	11.1
25–34	47	47.5
35–44	24	24.2
45–54	11	11.1
≥ 55	2	2.0
Missing	4	4.0
*Race*^*a*^		
American Indian/Alaska Native	57	57.6
Black	8	8.1
White	33	33.3
Other	11	11.1
*Ethnicity*		
Hispanic/Latino	18	18.2
Non-Hispanic/Latino	61	61.6
Missing	20	20.2
*Education*		
Did not graduate high school	30	30.3
High school diploma or GED	32	32.3
Trade or tech school	4	4.0
Some college	26	26.3
Bachelors or graduate degree	6	6.1
Missing	2	2.0
*Income*		
< $10,000	52	52.5
$10,000–$29,999	18	18.2
≥ $30,000	7	7.1
I don’t know	17	17.2
Missing	5	5.1
*Previous incarceration*		
Yes	62	62.6
No	33	33.3
Missing	3	3.0
*Length of incarceration (quartiles)*		
1–18 days	26	26.3
18–22 days	18	18.2
22–45 days	21	21.2
> 45 days	21	21.2
Missing	13	13.1
*General health*		
Excellent	6	6.1
Very good	22	22.2
Good	34	34.3
Fair	20	20.2
Poor	17	17.2
*Level of physical activity prior to incarceration*		
Very active	6	6.1
Somewhat active	6	6.1
Somewhat inactive	29	29.3
Very inactive	57	57.6
Missing	1	1.0
*Health condition*		
Asthma	19	19.2
Hypertention	8	8.1
High cholesterol	7	7.1
Diabetes or prediabetes	10	10.1
Overweight/obesity	61	61.7
Anxiety	64	64.7
Depression	61	61.6
Bipolar	28	28.3
Schizophrenia	8	8.1
PTSD	38	38.4
ADHD	11	11.1

Post-traumatic stress disorder (PTSD); attention deficit hyperactivity disorder (ADHD)

^a^Race categories are not mutually exclusive

Over 37% of women reported fair or poor general health and 57.6% of women reported being very inactive prior to incarceration (compared to 12.2% very or somewhat active, Table 1). Less than a quarter of women reported having asthma (19.2%), hypertension (8.1%), high cholesterol (7.1%), and/or diabetes or prediabetes (10.1%). More than half of women reported anxiety (64.7%) and/or depression (61.6%). Additionally, 28.3% of women reported being diagnosed with bipolar disorder, 8.1% with schizophrenia, 38.4% with PTSD, and 11.1% with ADHD.

### Rec-time

Of the 99 women who completed the questionnaire, 11.1% had never attended rec-time, 32.3% attended 1–2 times per week, 16.2% attended 3–4 times per week, and 38.4% attended every time it was offered (about 5 days a week, Table 2). Among participants who indicated they ever attended rec-time, 20.9% never exercised, 37.2% exercised 1–2 times per week, 12.8 exercised 3–4 times per week, and 27.9% exercised every time rec-time was offered (Table 2).

**Table 2:** Recreation time characteristics of women incarcerated at Coconino County Detention Facility, 2021 (n = 99)

	N	%
*Recreation time attendance*		
Never	11	11.1
1–2 times per week	32	32.3
3–4 times per week	16	16.2
Every time it is offered (about 5 days a week)	38	38.4
Missing	2	2.0
*Exercise during recreation time*^*a*^		
Never	18	20.9
1–2 times per week	32	37.2
3–4 times per week	11	12.8
Every time it is offered (about 5 days a week)	24	27.9
Missing	1	1.2

^a^Among the 86 participants who reported ever attending recreation time

### Rec-time facilitators

**Fig. 1:**
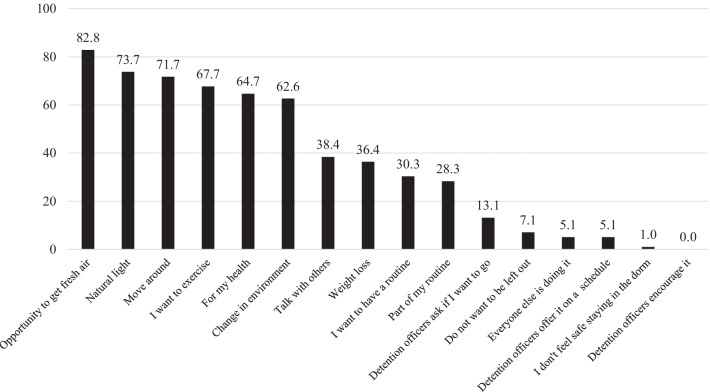
Facilitators to attending recreation time among women incarcerated at Coconino County Detention Facility, 2021 (n = 99). Women incarcerated at Coconino County were asked to indicate what motivated them or would motivate them to attend recreation time from a pre-determined list. Categories are not mutually exclusive as participants chose all that applied. Women were also given the opportunity to describe other facilitators to attending recreation time in short answer format

Most participants identified environmental- and health-related facilitators to attend rec-time (Fig. 1). The top two facilitators were an opportunity to get fresh air (82.8%) and natural light (73.7%). Additionally, 62.6% attended rec-time for a change in environment (compared to their housing units). Regarding health and exercise, many attended rec-time because they wanted to move around (71.7%), exercise (67.7%), for their health (64.7%), and weight loss (36.4%). Fewer participants indicated social reasons to attend rec-time including talking with others (38.4%), not wanting to be left out (7.1%), and everyone else was attending (5.1%). Fewer participants indicated they wanted a routine (30.3%), felt unsafe in the dorm (1.0%), or detention officers offered or encouraged attending rec-time (0%-13.1%).

Many women reiterated that attending rec-time was good for their mental health in some way. Facilitators to attend rec-time included:

Improve my depression.

Relief of anxieties.

…release my stress and to not think of everything going on in here and at home. It is my free mind time.

Mental health as a facilitator to attending recreation time was often accompanied by a discussion of spirituality or religion.

…People use rec-time to cope with depression and prayer outside also being outside you can yell and get your anger out by exercise instead of losing your insanity of no fresh air staying inside. Especially seeing and feeling sunlight is awesome.

To be able to pray for my family, kids, as I’m outside I feel my prayers are heard more outside, standing directly before the sun, I am reminding who I am […] a strong individual.

Play [racquetball] or just enjoy mother earth’s creations. Listen to the birds sing.

Being spiritual I find being outside helps me to interact with my God. The calmness of being alone outside helps me to find peace within.

Less often women indicated that it was just something to do, they liked to be alone, or get a tan.

When assessing differences in reported facilitators by length of incarceration, there were no differences. Alternatively, compared to women who had been previously incarcerated, women in jail for the first time were more likely to indicate “talking with others” as a facilitator (51.5% vs. 30.7%, p = 0.04) (see Additional file 2). There were no statistically significant differences in reported facilitators by whether a woman self-reported a physical or mental health condition (see Additional file 3). However, approaching statistical significance, a higher proportion of women with a physical health condition reported natural light as a facilitator (84.9% vs. 68.2% p = 0.07). Additionally, a higher proportion of women with a mental health condition reported opportunities to get fresh air (87.0% vs 73.3%, p = 0.1) and changes in environment (68.1% vs 50.0%, p = 0.09) as a facilitator to attending recreation time.

### Rec-time barriers

**Fig. 2:**
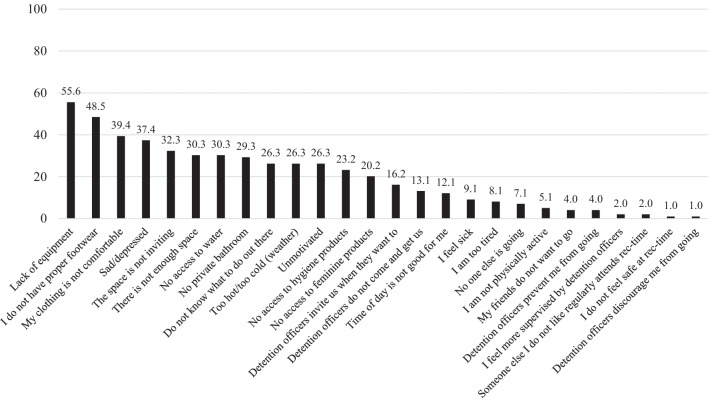
Barriers to attending recreation time among women incarcerated at coconino county detention facility, 2021 (n = 99). Women incarcerated at Coconino County were asked to indicate what prevented them or would prevent them from attending recreation time from a pre-determined list. Categories are not mutually exclusive as participants chose all that applied. Women were also given the opportunity to describe other facilitators to attending recreation time in short answer format

Most participants indicated an environment-, clothing-, or motivation-related barrier to attending rec-time (Fig. 2). Of the 99 participants, the environment, including the available equipment were barriers to attending rec-time including lack of equipment (55.6%); uninviting space (32.3%); lack of space (30.3%); no access to water (30.3%), a private bathroom (29.3%), hygiene products (23.2%), or feminine products (20.2%); or that it was too hot or too cold outside (26.3%). Women reiterated in an open-ended response that there was “*Not much out there…”* or that they “*Need more things to do”* while at rec-time. One woman also said that “*…it’s too depressing and plain looking.”*

Lack of proper footwear (48.5%) and no comfortable clothing (39.4%) were also common barriers to attending rec-time. One woman elaborated on the footwear saying, “*…the footwear is a biggie. It is dangerous to be in sandals.*” Another woman added that, “*They do not give extra underwear we would have to purchase extra and some of us don’t have the money.*”

Participants indicated being sad or depressed (37.4%), they were unmotivated (26.3), or they were sick or tired (8.1–9.1%) were barriers to attending rec-time. Some participants indicated they did not know what to do at rec-time (26.3%) or they were not physically active (5.1%). Social barriers to attending rec-time included no one else attending (7.1%), their friends did not want to attend (4.0%), or someone they did not like attended rec-time (2.0%). Finally, some participants indicated that detention officers were barriers as they did not invite them every time (16.2%), detention officers prevented them from attending (4.0%), they felt more supervised by detention officers at rec-time (2.0%), or detention officers discouraged them from attending (1.0%).

When assessing differences in reported barriers by length of incarceration, a higher proportion of women incarcerated longer than the median length of stay reported footwear (66.7% vs 31.8%, p = 0.001) and comfortable clothing (54.8% vs 25.0%, p = 0.005) compared to women who were incarcerated for less time than the median (see Additional file 2). A higher proportion of women who had a physical or mental health condition reported being sad or depressed as a barrier to attending recreation time compared to women who did not report a physical (54.5% vs 28.8%, p = 0.01) or mental health condition (46.4% vs 16.7%, p = 0.005). Women with a physical health condition reported not enough space (45.5% vs 25.8%, p = 0.04) or being unmotivated to attend recreation (42.4% vs 18.2%, p = 0.01) time as a barrier more often than women who did not have a physical health condition.

### Rec-time benefits

**Fig. 3:**
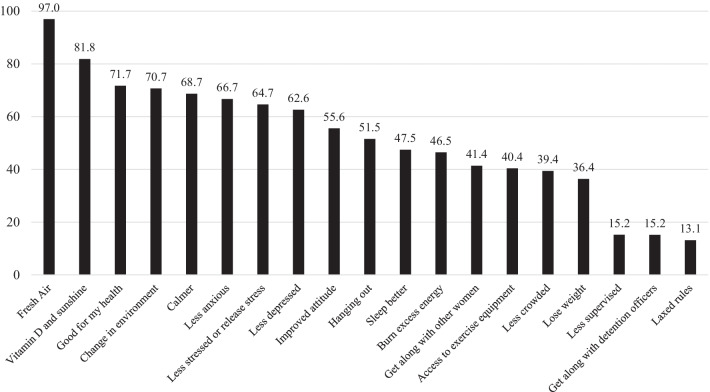
Benefits of attending recreation time at Coconino County detention facility. Women incarcerated at Coconino County were asked to indicate what personal benefits they received or would receive from attending recreation time from a pre-determined list. Categories are not mutually exclusive as participants chose all that applied. Women were also given the opportunity to describe other facilitators to attending recreation time in short answer format

Similar to facilitators and barriers, environment- and health-related perceived benefits of attending rec-time were reported more often than other benefits (Fig. 3). Most participants indicated that a change in environment (97.0%), vitamin D and sunshine (81.8%), and access to equipment (70.7%) were benefits of attending rec-time. Women believed that attending rec-time was also good for their health (71.7%); made them calmer (68.7%), less anxious (66.7%), less stressed (64.7%), and less depressed (62.6%); improved their attitude (55.6%) and sleep (47.5%); and helped them lose weight (36.4%). Women also indicated that hanging out at rec-time (51.5%) was a benefit that may lead to getting along with other women (41.4%) and detention officers (15.2%). Another benefit women brought up in open-ended responses were *“Passing [the] time”* and *“shortens my day”*.

When assessing differences in reported benefits by length of incarceration, there were no differences (see Additional file 2). Women who were in jail for the first time reported access to exercise equipment as a benefit more often than women who had been incarcerated before (57.6% vs 30.7%, p = 0.01). Women with a physical health condition reported sleeping better as a benefit of attending recreation time more often than women without a physical health condition (see Additional file 3). Finally, women with a mental health condition reported the change in environment (82.6% vs 43.3%, p < 0.0001) and reducing anxiety (75.4% vs 46.7%, p = 0.005), stress (71.0% vs 50.0%, p = 0.04), and depression (71.0% vs 43.3%, p = 0.009) as benefits more often that women without a mental health condition.

## Discussion

Our findings indicate that among women incarcerated in a rural county jail, most indicated environmental and health-related facilitators to attending rec-time. Women suggested that the environment, available equipment, clothing, and motivation were barriers to attending rec-time. Our findings have similarities and differences to the small body of research focused on facilitators and barriers to physical activity among incarcerated women.

Most surveyed women incarcerated at CCDF described environment-related facilitators and barriers to attending rec-time. During focus groups among women incarcerated in a maximum-security prison in the United States, women discussed lack of opportunities to be active even though there was a recreation department [24]. At CCDF, staff created a rec-time schedule around other programs and meals, limiting barriers to attending, unlike at the maximum-security prison. However, the maximum-security prison offered fitness classes, which may require accommodating an outside employee’s schedule. Among older women (50 years and older) incarcerated in prisons in Switzerland, women reported that there were physical activity spaces and opportunities available to them [23]. Age was considered a barrier to physical activity. Women indicated available activities while in prison, such as volleyball, were more appropriate for younger women incarcerated. Compared to women incarcerated in prisons in Switzerland, timing of rec-time was not described as a barrier among women incarcerated at CCDF. Similar to women in prisons in Switzerland, lack of equipment and not knowing what to do in the recreation space were common barriers among women incarcerated at CCDF. The limitations of appropriate activities available during recreation time among women incarcerated may be important and potentially easily addressable barriers to attending and being physically active during recreation time. Future research should determine physical activities that women would enjoy and motivate them to attend recreation time.

Older women incarcerated in prisons in Switzerland indicated they do not exercise because they did not want to or were not healthy enough to engage in physical activity [23]. Women believed that the sedentary lifestyle in prison was associated with their health problems. About 2% of our sample were 55 years and older. However, a large proportion of women reported health conditions that may impact a woman’s self-efficacy and belief they can participate in physical activity. Additionally, women incarcerated at CCDF discussed mental health as a barrier and rec-time being good for their health and made them less anxious, stressed, and depressed as benefits of rec-time. Similarly, women incarcerated in a maximum-security prison mentioned that a sedentary lifestyle in prison leads to more complications down the road [24]. Motivation, self-efficacy, and the capacity to execute physical activity safely and effectively is impacted by many things, including how physically active someone is before being incarcerated, mental health, physical health, and the environment in which someone participates in physical activity. Creating an environment in jails that provides opportunities to not only be physically active but also learn ways to be physically active is essential to reduce barriers and improve self-efficacy. This may include structured physical activity programs or guidance on physical activities in their space such as instructions on equipment and physical activity materials. However, environmental changes alone may not improve motivation to be physically active. Previous research has shown that face-to-face interventions, goal settings, self-monitoring programs have improved motivation and should be explored in the jail setting [32].

In contrast to our findings, women incarcerated in a maximum security prison mentioned that staff did not promote in-cell exercise [24]. Although we did not specifically ask about all staff at CCDF, only a small proportion of women suggested staff prevented or discouraged women from attending rec-time. Though, many participants did suggest that staff should improve practices to encourage rec-time attendance. Finally, a common theme among women incarcerated in prison and CCDF was that issued sandals are not conducive to being physically active. Surveyed women at CCDF believed that lack of proper footwear was a barrier to attending rec-time saying that it may even be dangerous. Footwear is important for comfort and safety. All individuals, when booked into the detention facility, are provided a pair of sandals. Sneakers are not available unless purchased through the commissary. To note, in our previous research, we found that 92% of women wore facility-issued sandals and only 2% wore purchased sneakers [31]. Inappropriate clothing and footwear may be a safety issue when participating in physical activities in any environment. Jails do not provide sneakers or alternative clothing to participate in physical activities unless purchased from the commissary. Most women do not have an income while incarcerated and, in our study sample, over half of women had an income of less than $10,000 per year; thus, purchasing shoes and clothing to participate in physical activities while incarcerated may not be a priority.

Previous studies did not assess differences in barriers or facilitators to being physically active, which may be important for developing targeted physical activity interventions or programs. Although we found few differences in reported facilitators, barriers, and benefits by length of incarceration, previous incarcerations, or physical or mental health conditions, we found that women with self-reported mental health conditions reported more benefits to attending recreation time, specifically that attending recreation time contributed to less anxiety, stress, and depression. To note, we were limited to a small sample size of 99. We may have found more differences by characteristics of women incarcerated with a larger sample. Additionally, physical and mental health conditions were limited to self-reported conditions that had been diagnosed by a health care professional, which may contribute to misclassification of women either with or without a mental or physical health condition.

We asked participants about facilitators and barriers to attending rec-time, not physical activity. Other studies also discussed opportunities to being physically active outside of rec-time. Additionally, previous findings were obtained from focus groups [23, 24]. The original intent of our research was to host focus group discussions among women incarcerated at CCDF to understand the barriers and facilitators to attending rec-time. However, due to the COVID-19 pandemic, CCDF was closed to outside visitors, programs, and researchers; thus, the topics of the focus group guide were transformed into a questionnaire. Future research would benefit from qualitative discussions with women to clarify and provide more context regarding their views on the benefits and barriers to being physically active while incarcerated.

A high proportion of women (almost 90%) incarcerated at CCDF self-reported ever attending rec-time and of those women, almost 80% indicated they exercised during rec-time at least 1–2 times per week. This is a substantially more active sample of women compared to previous findings in the same detention facility. Previous studies did not rely on self-report of physical activity, which introduces self-reporting bias in which women overreport their physical activity levels among the general population [33]. Thus, using objective observational methods, women were found to engage in recreation time and physical activity less often. Only 25% of women incarcerated at CCDF attended rec-time while almost 60% of women who did attend were sedentary during rec-time [22]. To note, if the current study sample of women incarcerated in jail is more active than other groups of women incarcerated, barriers and facilitators to being physically active may differ among less-active women. There may be other explanations and limitations that explain the discrepancy between our current study and previous research at the same facility. The COVID-19 pandemic limited our research as not everyone incarcerated had access to rec-time. CCDF required all individuals booked into the jail to be quarantined for a 14-day period in which rec-time was not available. Thus, our sample of women had been quarantined, without access to rec-time, prior to participating in our study. Women indicated that “*They put us on a 14-day lock down before we can do anything*”. Women may have been more likely to want to attend rec-time after being quarantined compared to before the COVID-19 pandemic. Similarly, our sample was limited to women who were incarcerated 14 days or longer. Most individuals are released from jail prior to reaching the full quarantine period [34]; thus, we did not have the opportunity to receive information from women who were incarcerated for shorter amounts of time. Only recruiting women who had completed a 14-day quarantine period may limit generalizability and introduce sampling bias into our study. Additionally, because findings were self-reported, higher rates of attendance and exercise may be due to the nature of overreporting physical activity-related behaviors [33, 35]. In a 2019 study among individuals incarcerated in jail, 70% self-reported ever attending rec-time [30], a much higher proportion compared to objectively measured attendance.

## Conclusions

Women have different health needs compared to men and the rates of women’s incarceration are increasing, especially in Arizona. As health and environment are important facilitators and barriers to being physically active among women in jail, it is important to identify appropriate environmental and policy interventions to increase the use of rec-time and physical activity. Because women perceive health as a facilitator and benefit for attending rec-time, rec-time may have larger implications for correctional facilities such as improved mental and physical health of individuals incarcerated in the facilities. First, if a correctional facility does not offer rec-time or a similar alternative, one should be established and accessible as “at a minimum… staff shall provide the pretrial inmate with… one hour daily of outside recreation, weather permitting” (28 CFR § 551.115) and not providing rec-time may be considered cruel and unusual punishment [36]. Second, creating an environment that is welcoming and conducive to being physically active is imperative. Creating an environment for women to be more physically active may include green-space, mats or softer flooring, or more accessible equipment. Third, to improve safety, sneakers should be provided at no cost as sandals were considered a barrier to attending rec-time and being physically active, even unsafe. Finally, women not knowing what to do during rec-time indicates that women may benefit from more structured physical activity programs or guidance on physical activities in their space such as instructions on equipment and physical activity materials.

## Supplementary Information

**Additional file 1:** Questionnaire distributed to women incarcerated at Coconino County Detention Facility.

**Additional file 2:** Differences in facilitators, barriers, and benefits of attending recreation time among women incarcerated at Coconino County Detention Facility, by length of incarceration and whether a woman was previously incarcerated.

**Additional file 3:** Differences in facilitators, barriers, and benefits of attending recreation time among women incarcerated at Coconino County Detention Facility, by whether a woman had a physical or mental health condition.

## Data Availability

The datasets generated and/or analyzed during the current study are not publicly available because of agreements between Northern Arizona University and Coconino County Detention Facility but are available from the corresponding author on reasonable request.

## Abbreviations

CCDF: Coconino County Detention Facility
PTSD: Post traumatic stress disorder
ADHD: Attention deficit hyperactivity disorder
