# Supplementary material for: Barriers and facilitators to attending and being physically active during recreation time among women incarcerated
Source: BMC Womens Health. 2022 Jun 17;22:239. doi: 10.1186/s12905-022-01831-w (PMC9205544; doi:10.1186/s12905-022-01831-w)
Supplement: Supplementary file 1 — Additional file 1: Questionnaire distributed to women incarcerated at Coconino County Detention Facility. [file 12905_2022_1831_MOESM1_ESM.pdf]

Additional File 1. Questionnaire

Rec-Time

First, we have questions about rec-time. At CCDF you are given an opportunity daily to attend what is called recreation time (or rec-time) outside for up to an hour.

Here is a picture of rec-time spaces available to women at CCDF.

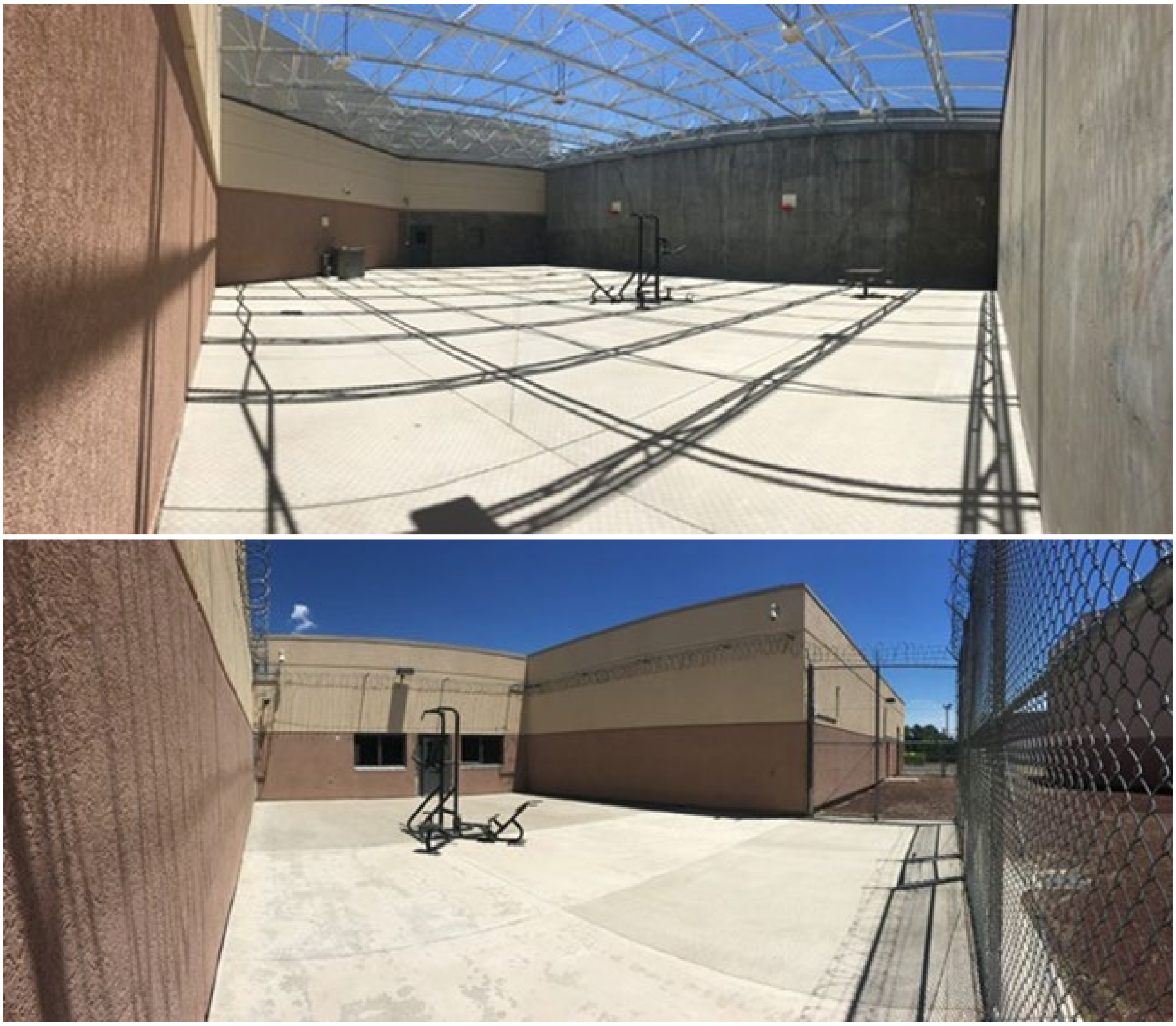

1. Have you ever attended rec-time at CCDF?
   - ◻ I have never attended rec-time and do not want to attend
   - ◻ I have never attended rec-time, but I want to attend
   - ◻ I have attended rec-time

2. How often *do you attend* rec-time each week?
   - ◻ Every time it is offered (about 5 days a week)
   - ◻ 3-4 times a week

- ◻ 1-2 times a week
- ◻ Never

3. How often *do you exercise* at rec-time each week?
   - ◻ Every time it is offered (about 5 days a week)
   - ◻ 3-4 times a week
   - ◻ 1-2 times a week
   - ◻ Never

4. *What motivates you* or would motivate you to attend rec-time? (Check all that apply)
   - ◻ Opportunity to get fresh air
   - ◻ Natural light
   - ◻ Change in environment
   - ◻ Move around
   - ◻ Talk with others
   - ◻ Others attend/don't want to be left out
   - ◻ Everyone else is doing it
   - ◻ I don't feel safe staying in the dorm during rec-time
   - ◻ I want to exercise
   - ◻ Part of your routine
   - ◻ I want to have a routine
   - ◻ For my health
   - ◻ Weight loss
   - ◻ Detention officer encourages it
   - ◻ Detention officer asks if I want to go
   - ◻ Detention officers offer it on a consistent schedule
   - ◻ Other:____

   ____

   ____

5. What are your *top three motivations* for attending rec-time? You may choose from the list above or add your own.
   1. ________________________
   2. ________________________
   3. ________________________

6. Please select the option(s) that best describe why you have not or would not attend rec-time. (Check all that apply)
   - ◻ I don't feel safe at rec-time
   - ◻ The space is not inviting
   - ◻ There is not enough space
   - ◻ Lack of equipment
   - ◻ Don't know what to do out there
   - ◻ I'm not physically active
   - ◻ My friends don't want to go
   - ◻ My clothing isn't comfortable
   - ◻ I don't have proper footwear
   - ◻ No one else is going
   - ◻ Detention officers do not come and get us
   - ◻ Detention officers only invite us when they want to
   - ◻ Detention officers discourage me from going
   - ◻ Detention officers prevent me from going
   - ◻ I feel more supervised by detention officers
   - ◻ Too cold/too hot (weather)
   - ◻ Time of day not good for me
   - ◻ No access to hygiene products
   - ◻ No access to feminine products
   - ◻ No access to water while at rec time
   - ◻ No private bathroom
   - ◻ I am too tired
   - ◻ I feel sick
   - ◻ Unmotivated
   - ◻ Sad/depressed
   - ◻ Someone else that I don't like regularly attends rec-time
   - ◻ Other:____

   ____

   ____

7. What activities do you like to do during rec-time? (Check all that apply)

- ◻ Walk
- ◻ Jog or run
- ◻ Stretch
- ◻ Use exercise equipment
- ◻ Pair/group workouts
- ◻ Yoga
- ◻ Pushups, sit-ups, leg lifts, planks
- ◻ Sunbathe
- ◻ Talk to others
- ◻ Reflection
- ◻ Meditate
- ◻ Prayer
- ◻ Change in environment/scenery
- ◻ I have never attended
- ◻ Other:____

____

8. For you personally, what benefits *do you think* result from attending rec-time? (Check all that apply)
   - □ Vitamin D/Sunshine
   - □ Fresh air
   - □ Change in environment/scenery
   - □ Access to exercise equipment
   - □ Less crowded
   - □ Less supervised (not being watched as closely)
   - □ Laxed rules
   - □ Hanging out
   - □ Calmer
   - □ Less anxious
   - □ Less depressed
   - □ Sleep better
   - □ Improved attitude
   - □ Less stressed / Release stress
   - □ Lose weight
   - □ Good for my health
   - □ Burn excess energy
   - □ Get along with detention officers
   - □ Get along with other women
   - □ Other:____

   ____

9. For you personally, what are the *top three benefits* of attending rec-time? You may choose from the list above or add your own.
   1. ___________________________
   2. ___________________________
   3. ___________________________

10. Detention officers encourage women to attend rec-time.

- ◻ Strongly agree
- ◻ Agree
- ◻ Somewhat agree
- ◻ Somewhat disagree
- ◻ Disagree
- ◻ Strongly disagree

11. Detention officer *actions* impact whether or not women attend rec-time.

- ◻ Strongly agree
- ◻ Agree
- ◻ Somewhat agree
- ◻ Somewhat disagree
- ◻ Disagree
- ◻ Strongly disagree

12. Detention officer *words* impact whether or not women attend rec-time.

- ◻ Strongly agree
- ◻ Agree
- ◻ Somewhat agree
- ◻ Somewhat disagree
- ◻ Disagree
- ◻ Strongly disagree

13. Detention officer *actions* impact whether or not women exercise during rec-time.

- ◻ Strongly agree
- ◻ Agree
- ◻ Somewhat agree
- ◻ Somewhat disagree
- ◻ Disagree
- ◻ Strongly disagree

14. Detention officer *words* impact whether or not women exercise during rec-time.

- ◻ Strongly agree
- ◻ Agree
- ◻ Somewhat agree
- ◻ Somewhat disagree
- ◻ Disagree
- ◻ Strongly disagree

15. Detention officers *encourage* women to attend rec-time by doing the following:
____
____
____

16. Detention officers *discourage* women to attend rec-time by doing the following:
____
____
____

17. If the *CCDF administrators* wanted to make rec-time better, what do you think they should do? (Check all that apply)
    - □ Provide exercise instructions so we know how to use the equipment
    - □ Provide exercise instruction for general exercise ideas
    - □ Provide exercise classes (like examples below)
        - □ Yoga class
        - □ Zumba class
        - □ Aerobics class
        - □ Tai chi class
        - □ Circuit Training
    - □ Provide more exercise equipment
    - □ Update exercise equipment
    - □ Add plants or other greenery
    - □ Paint murals on walls
    - □ Add more places to sit
    - □ Other ideas: ____

    ____
    ____

18. If the *detention officers* wanted to make rec-time better, what do you think they should do? (Check all that apply)
    - □ Encourage women to attend rec-time more
    - □ Keep a consistent schedule
    - □ Be flexible on times for attendance
    - □ Be flexible on length of rec-time
    - □ Be flexible on granting requests during rec-time (for example, allowing water breaks, retrieving jackets, retrieving hygiene products)
    - □ Other ideas: ____

    ____
    ____

19. Is there anything else about rec-time and exercising at CCDF that we should know?

____
____
____
____
____

Exercise in the Dorm

Now, we would like to learn more about your exercise habits in your dorm at CCDF.

20. Do you exercise in your dorm/pod?

- ◻ Yes
- ◻ No

21. If you exercise in your dorm, what ones do you do?

____
____
____
____

22. How often do you exercise in your dorm?

- ◻ 5 or more days a week
- ◻ 3-4 times a week
- ◻ 1-2 times a week
- ◻ Never or almost never

23. Do you generally exercise more in your dorm or at rec-time?

- ◻ I do not exercise
- ◻ I exercise more in my dorm than at rec-time
- ◻ I exercise more at rec-time than in my dorm
- ◻ I exercise about the same in my dorm and at rec-time

COVID-19

Now, we would like to better understand your thoughts and experiences related to the COVID-19 pandemic.

24. Have you ever been diagnosed with COVID-19?
    - □ Yes
    - □ No

25. Have you been diagnosed with COVID-19 during your stay at CCDF?
    - □ Yes
    - □ No

26. Have you been tested for COVID-19 at CCDF?
    - □ Yes
    - □ No

27. Has COVID-19 affected your rec-time attendance?
    - □ Yes
    - □ No

28. Has COVID-19 affected your exercise at CCDF?
    - □ Yes
    - □ No

29. If you said COVID-19 has affected your rec-time attendance or exercise at CCDF, please describe how COVID-19 has affected your exercise and/or rec-time attendance?

    ____
    ____
    ____
    ____
    ____
    ____

Health Questions

Next, we want to learn about your general physical and mental health.

30. In general, would you say that your health is excellent, very good, good, fair, or poor?
    - ☐ Excellent
    - ☐ Very good
    - ☐ Good
    - ☐ Fair
    - ☐ Poor

31. How active were you in your day-to-day life before your current incarceration at CCDF (including for work, travel, and exercise)?
    - ☐ Very active
    - ☐ Somewhat active
    - ☐ Somewhat inactive
    - ☐ Very inactive

32. How tall are you?

    Feet _____

    Inches _______

33. How much do you weigh?

    Pounds ______

34. Has a doctor or other health professional ever told you that you have any of the following? (Check all that apply)
    - ☐ Asthma
    - ☐ Hypertension (also called high blood pressure)
    - ☐ High cholesterol
    - ☐ Diabetes (also called sugar diabetes)
    - ☐ Prediabetes
    - ☐ Anxiety
    - ☐ Depression
    - ☐ Bipolar disorder or manic depression
    - ☐ Schizophrenia
    - ☐ Post-traumatic stress disorder (PTSD)
    - ☐ Attention deficit hyperactivity disorder (ADD or ADHD)

35. Do you self-identify as having a disability?
    - □ Yes
    - □ No

36. When you were in school, did you get special education services?
    - □ Yes
    - □ No

37. Are you or do you have serious difficulty hearing?
    - □ Yes
    - □ No

38. Are you blind or do you have serious difficulty seeing, even when wearing glasses?
    - □ Yes
    - □ No

39. Because of a physical, mental, or emotional condition, do you have serious difficulty concentrating, remembering, or making decisions?
    - □ Yes
    - □ No

40. Do you have serious difficulty walking or climbing stairs?
    - □ Yes
    - □ No

41. Do you have difficulty dressing or bathing?
    - □ Yes
    - □ No

42. Because of a physical, mental, or emotional condition, do you have difficulty doing errands alone such as visiting a doctor's office or shopping?
    - □ Yes
    - □ No

43. Does your disability restrict you from attending rec-time like other incarcerated women at CCDF?
    - □ Yes
    - □ No
    - □ I do not have a disability

44. If you self-identify as having a disability, do you receive accommodations regarding attending or utilizing rec-time?
    - □ Yes
    - □ No
    - □ I do not have a disability

45. If you receive accommodations for rec-time, what accommodations do you receive?
    ____
    ____
    ____
    ____

46. If you receive accommodations for your disability to attend or utilize rec-time, are your accommodations always provided?
    - □ Yes
    - □ No
    - □ I do not have a disability

General Information

Finally, we will ask you general questions about yourself.

47. What Pod are you currently in?
   - ☐ A
   - ☐ B
   - ☐ C
   - ☐ F

48. When were you booked into the CCDF?
   Day_________
   Month________
   Year_________

49. Before this incarceration, have you been previously incarcerated at CCDF?
   - ☐ Yes
   - ☐ No

50. What is your age?
   - ☐ 18-24
   - ☐ 25-34
   - ☐ 35-44
   - ☐ 45-54
   - ☐ 55-64
   - ☐ 65 or older

51. What is your race? (Check all that apply)
   - ☐ American Indian or Alaskan Native
   - ☐ Asian
   - ☐ Native Hawaiian and Pacific Islander
   - ☐ Black/African American
   - ☐ White
   - ☐ Other Race: _________________________

52. What is your ethnicity?
   - ☐ Hispanic/Latina
   - ☐ Non-Hispanic/Latina

53. What is your education level?

- □ Did not graduate high school
- □ High school diploma or GED
- □ Trade or tech school
- □ Some college
- □ 4-year college degree
- □ Graduate degree

54. What was your household income level before you were incarcerated?

- □ $0 – $9,999
- □ $10,000 – $29,999
- □ $30,000 – $39,999
- □ $40,000 – 49,999
- □ Greater than or equal to 50,000
- □ I do not know

This is the end of the survey. Thank you very much for your participation. Place the completed survey back in the brown envelope and seal it. Give the completed survey sealed in the brown envelope back to the staff member when they return. You will then receive a white envelope with incentives from the staff member.
