# Supplementary material for: Barriers and facilitators to attending and being physically active during recreation time among women incarcerated
Source: BMC Womens Health. 2022 Jun 17;22:239. doi: 10.1186/s12905-022-01831-w (PMC9205544; doi:10.1186/s12905-022-01831-w)
Supplement: Supplementary file 2 — Additional file 2: Differences in facilitators, barriers, and benefits of attending recreation time among women incarcerated at Coconino County Detention Facility, by length of incarceration and whether a woman was previously incarcerated. [file 12905_2022_1831_MOESM2_ESM.docx]

| Additional File 2. Differences in facilitators, barriers, and benefits of attending recreation time among women incarcerated at Coconino County Detention Facility, by length of incarceration and whether a woman was previously incarcerated, 2021 | | | | | | | | | | | | | | | | | | |
| --- | --- | --- | --- | --- | --- | --- | --- | --- | --- | --- | --- | --- | --- | --- | --- | --- | --- | --- |
|  | Overall (n = 99) | Length of Incarceration | | | | | | | Previous Incarceration | | | | | | | | |  |
|  |  | ≤ Median ^a^  (n = 44) | | > Median ^a^  (n = 42) | |  | | No Previous Incarceration (n = 33) | | | | Previous Incarceration  (n = 62) | | |  | |  |  |
|  | % | n | % | n | % | p ^b^ | n | | | % | n | | % | p ^b^ | |  |  |  |
| **Facilitators** |  |  |  |  |  |  |  | | |  |  | |  |  | |  |  |  |
| Opportunity to get fresh air | 82.8 | 37 | 84.1 | 34 | 39.5 | 0.7 | 29 | | | 87.9 | 49 | | 79.0 | 0.3 | |  |  |  |
| Natural light | 73.7 | 32 | 72.7 | 31 | 73.8 | 0.9 | 26 | | | 78.8 | 44 | | 71.0 | 0.4 | |  |  |  |
| Move around | 71.7 | 32 | 72.7 | 30 | 71.4 | 0.9 | 23 | | | 69.7 | 44 | | 71.0 | 0.9 | |  |  |  |
| I want to exercise | 67.7 | 28 | 63.6 | 29 | 69.1 | 0.6 | 22 | | | 66.7 | 41 | | 66.1 | >0.9 | |  |  |  |
| For my health | 64.7 | 28 | 63.6 | 28 | 66.7 | 0.8 | 21 | | | 63.6 | 40 | | 64.5 | >0.9 | |  |  |  |
| Change in environment | 62.6 | 27 | 61.4 | 25 | 59.5 | 0.9 | 23 | | | 69.7 | 36 | | 58.1 | 0.3 | |  |  |  |
| Talk with others | 38.4 | 16 | 36.4 | 17 | 40.5 | 0.7 | **17** | | | **51.5** | **19** | | **30.7** | **0.04** | |  |  |  |
| Weight loss | 36.4 | 16 | 36.4 | 13 | 31.0 | 0.6 | 12 | | | 36.4 | 21 | | 33.9 | 0.8 | |  |  |  |
| I want to have a routine | 30.3 | 13 | 39.4 | 16 | 25.8 | 0.3 | 13 | | | 39.4 | 16 | | 25.8 | 0.2 | |  |  |  |
| Part of my routine | 28.3 | 13 | 29.6 | 10 | 23.8 | 0.5 | 10 | | | 30.3 | 17 | | 27.4 | 0.8 | |  |  |  |
| Detention officers ask if I want to go | 13.1 | 5 | 11.4 | 4 | 9.5 | 0.8 | 5 | | | 15.2 | 8 | | 12.9 | 0.8 | |  |  |  |
|  |  |  |  |  |  |  |  | | |  |  | |  |  | |  |  |  |
| **Barriers** |  |  |  |  |  |  |  | | |  |  | |  |  | |  |  |  |
| Lack of equipment | 55.6 | 25 | 56.8 | 27 | 64.3 | 0.5 | 22 | | | 66.7 | 31 | | 50.0 | 0.1 | |  |  |  |
| I do not have proper footwear | 48.5 | **14** | **31.8** | **28** | **66.7** | **0.001** | 18 | | | 54.6 | 28 | | 45.2 | 0.4 | |  |  |  |
| My clothing is not comfortable | 39.4 | **11** | **25.0** | **23** | **54.8** | **0.005** | 14 | | | 42.4 | 22 | | 35.5 | 0.5 | |  |  |  |
| Sad/depressed | 37.4 | 14 | 31.8 | 19 | 45.2 | 0.2 | 14 | | | 42.4 | 21 | | 33.9 | 0.4 | |  |  |  |
| The space is not inviting | 32.3 | 14 | 31.8 | 15 | 35.7 | 0.7 | 12 | | | 36.4 | 16 | | 25.8 | 0.3 | |  |  |  |
| There is not enough space | 30.3 | 12 | 27.3 | 16 | 38.1 | 0.3 | 11 | | | 33.3 | 20 | | 32.3 | 0.9 | |  |  |  |
| No access to water | 30.3 | 14 | 31.8 | 14 | 33.3 | 0.9 | 7 | | | 21.2 | 23 | | 37.1 | 0.1 | |  |  |  |
| No private bathroom | 29.3 | 10 | 22.7 | 16 | 38.1 | 0.1 | 10 | | | 30.3 | 18 | | 29.0 | 0.9 | |  |  |  |
| Do not know what to do out there | 26.3 | 12 | 27.3 | 10 | 23.8 | 0.7 | 9 | | | 27.3 | 16 | | 25.8 | 0.9 | |  |  |  |
| Too hot/too cold (weather) | 26.3 | 12 | 27.3 | 12 | 28.6 | 0.9 | 9 | | | 27.3 | 16 | | 25.8 | 0.9 | |  |  |  |
| Unmotivated | 26.3 | 10 | 22.7 | 13 | 31.0 | 0.4 | 9 | | | 27.3 | 15 | | 24.2 | 0.7 | |  |  |  |
| No access to hygiene products | 23.2 | 6 | 13.6 | 12 | 28.6 | 0.09 | 9 | | | 27.3 | 14 | | 22.6 | 0.6 | |  |  |  |
| No access to feminine products | 20.2 | 6 | 13.6 | 11 | 26.2 | 0.1 | 5 | | | 15.2 | 15 | | 24.6 | 0.3 | |  |  |  |
| Detention officers invite us when they want to | 16.2 | 4 | 9.1 | 10 | 23.8 | 0.06 | 7 | | | 21.2 | 9 | | 14.5 | 0.4 | |  |  |  |
| Detention officers do not come and get us | 13.1 | 3 | 6.8 | 9 | 21.4 | 0.05 | 6 | | | 18.2 | 7 | | 11.3 | 04 | |  |  |  |
| Time of day is not good for me | 12.1 | **2** | **4.6** | **10** | **23.8** | **0.01** | 4 | | | 12.1 | 8 | | 12.9 | >0.9 | |  |  |  |
|  |  |  |  |  |  |  |  | | |  |  | |  |  | |  |  |  |
| **Benefits** |  |  |  |  |  |  |  | | |  |  | |  |  | |  |  |  |
| Fresh air | 97.0 | 43 | 97.7 | 41 | 97.6 | >0.9 | 32 | | | 97.0 | 60 | | 96.8 | >0.9 | |  |  |  |
| Vitamin D and Sunshine | 81.8 | 34 | 77.3 | 37 | 88.1 | 0.2 | 22 | | | 66.7 | 45 | | 72.6 | 0.5 | |  |  |  |
| Good for my health | 71.7 | 31 | 70.5 | 31 | 73.8 | 0.7 | 29 | | | 87.9 | 48 | | 77.4 | 0.2 | |  |  |  |
| Change in environment | 70.7 | 32 | 72.7 | 30 | 71.4 | 0.9 | 23 | | | 69.7 | 47 | | 75.8 | 0.5 | |  |  |  |
| Calmer | 68.7 | 31 | 70.5 | 29 | 69.1 | 0.9 | 24 | | | 72.7 | 43 | | 69.4 | 0.7 | |  |  |  |
| Less anxious | 66.7 | 29 | 65.9 | 28 | 66.7 | 0.9 | 23 | | | 69.7 | 42 | | 67.7 | 0.8 | |  |  |  |
| Less stressed or release stress | 64.7 | 29 | 65.9 | 26 | 61.9 | 0.7 | 22 | | | 66.7 | 38 | | 61.3 | 0.6 | |  |  |  |
| Less depressed | 62.6 | 27 | 61.4 | 25 | 59.5 | 0.9 | 20 | | | 60.6 | 39 | | 62.9 | 0.8 | |  |  |  |
| Improved attitude | 55.6 | 25 | 56.8 | 23 | 54.8 | 0.8 | 18 | | | 54.6 | 36 | | 58.1 | 0.7 | |  |  |  |
| Hanging out | 51.5 | 23 | 52.3 | 19 | 45.2 | 0.5 | 14 | | | 42.4 | 34 | | 54.8 | 0.2 | |  |  |  |
| Sleep better | 47.5 | 20 | 45.5 | 19 | 45.2 | >0.9 | 16 | | | 48.5 | 29 | | 46.8 | 0.9 | |  |  |  |
| Burn excess energy | 46.5 | 19 | 43.2 | 20 | 47.6 | 0.7 | 14 | | | 42.4 | 26 | | 41.9 | >0.9 | |  |  |  |
| Get along with other women | 41.4 | 15 | 34.1 | 22 | 52.4 | 0.09 | 16 | | | 48.5 | 29 | | 46.7 | >0.9 | |  |  |  |
| Access to exercise equipment | 40.4 | 16 | 36.4 | 18 | 42.9 | 0.5 | **19** | | | **57.6** | **19** | | **30.7** | **0.01** | |  |  |  |
| Less crowded | 40.4 | 14 | 31.8 | 20 | 47.6 | 0.1 | 12 | | | 36.4 | 25 | | 40.3 | 0.7 | |  |  |  |
| Lose weight | 36.4 | 18 | 40.9 | 11 | 26.2 | 0.1 | 13 | | | 39.4 | 22 | | 35.5 | 0.7 | |  |  |  |
| Less supervised | 15.2 | 5 | 11.4 | 8 | 19.1 | 0.3 | 4 | | | 12.1 | 10 | | 16.1 | 0.6 | |  |  |  |
| Get along with detention officers | 15.2 | 5 | 11.4 | 6 | 14.3 | 0.7 | 3 | | | 9.1 | 11 | | 17.7 | 0.3 | |  |  |  |
| Laxed rules | 13.1 | 6 | 13.6 | 6 | 14.3 | >0.9 | 7 | | | 21.2 | 5 | | 8.1 | 0.07 | |  |  |  |
| ^a^ Median length of incarceration = 22 days  ^b^ Group differences in responses were assessed using chi square tests  Facilitators with less than 10 respondents: Do not want to be left out; Everyone else is doing it; Detention officers offer it on a schedule; I don’t feel safe staying in the dorm; Detention officers encourage it  Barriers with less than 10 respondents: I feel sick; I am too tired; No one else is going; I am not physically active; My friends do not want to go  Detention officers prevent me from going; I feel more supervised by detention officers; Someone else I do not like regularly attends rec-time; I do not feel safe at rec-time; Detention officers discourage me from going  There were no benefits with less than 10 respondents | | | | | | | | | | | | | | | | | | |
