# Supplementary material for: Barriers and facilitators to attending and being physically active during recreation time among women incarcerated
Source: BMC Womens Health. 2022 Jun 17;22:239. doi: 10.1186/s12905-022-01831-w (PMC9205544; doi:10.1186/s12905-022-01831-w)
Supplement: Supplementary file 3 — Additional file 3: Differences in facilitators, barriers, and benefits of attending recreation time among women incarcerated at Coconino County Detention Facility, by whether a woman had a physical or mental health condition. [file 12905_2022_1831_MOESM3_ESM.docx]

| Additional File 3. Differences in facilitators, barriers, and benefits of attending recreation time among women incarcerated at Coconino County Detention Facility, by whether a woman had a physical or mental health condition, 2021 | | | | | | | | | | | | |
| --- | --- | --- | --- | --- | --- | --- | --- | --- | --- | --- | --- | --- |
|  | Overall (n = 99) | Physical Health Condition ^a^ | | | | | Mental Health Condition ^b^ | | | | | |
|  |  | No  (n = 66) | | Yes  (n = 33) | |  | No  (n = 30) | | Yes  (n = 69) | | |  |
|  | % | n | % | n | % | p ^c^ | n | % | n | % | p ^c^ | |
| **Facilitators** |  |  |  |  |  |  |  |  |  |  |  | |
| Opportunity to get fresh air | 82.8 | 53 | 80.3 | 29 | 87.9 | 0.3 | 22 | 73.3 | 60 | 87.0 | 0.1 | |
| Natural light | 73.7 | 45 | 68.2 | 28 | 84.9 | 0.07 | 20 | 66.7 | 53 | 76.8 | 0.3 | |
| Move around | 71.7 | 46 | 69.7 | 25 | 75.8 | 0.5 | 20 | 66.7 | 51 | 73.9 | 0.5 | |
| I want to exercise | 67.7 | 44 | 66.7 | 23 | 69.7 | 0.7 | 22 | 73.3 | 45 | 65.2 | 0.4 | |
| For my health | 64.7 | 42 | 63.6 | 22 | 66.7 | 0.8 | 20 | 66.7 | 44 | 63.8 | 0.8 | |
| Change in environment | 62.6 | 39 | 59.1 | 23 | 69.7 | 0.3 | 15 | 50.0 | 47 | 68.1 | 0.09 | |
| Talk with others | 38.4 | 24 | 36.4 | 14 | 42.4 | 0.6 | 9 | 30.0 | 29 | 42.0 | 0.3 | |
| Weight loss | 36.4 | 24 | 36.4 | 12 | 36.4 | >0.9 | 11 | 36.7 | 25 | 36.2 | >0.9 | |
| I want to have a routine | 30.3 | 21 | 31.8 | 9 | 27.3 | 0.6 | 10 | 33.3 | 20 | 29.0 | 0.7 | |
| Part of my routine | 28.3 | 18 | 27.3 | 10 | 30.3 | 0.8 | 8 | 26.7 | 20 | 29.0 | 0.8 | |
| Detention officers ask if I want to go | 13.1 | 8 | 12.1 | 5 | 15.2 | 0.7 | 5 | 16.7 | 8 | 11.6 | 0.5 | |
|  |  |  |  |  |  |  |  |  |  |  |  | |
| **Barriers** |  |  |  |  |  |  |  |  |  |  |  | |
| Lack of equipment | 55.6 | 33 | 50.0 | 22 | 66.7 | 0.1 | 15 | 50.0 | 40 | 58.0 | 0.5 | |
| I do not have proper footwear | 48.5 | 31 | 47.0 | 17 | 51.5 | 0.7 | 14 | 46.7 | 34 | 49.3 | 0.8 | |
| My clothing is not comfortable | 39.4 | 28 | 42.4 | 11 | 33.3 | 0.4 | 13 | 43.3 | 26 | 37.7 | 0.6 | |
| Sad/depressed | 37.4 | **19** | **28.8** | **18** | **54.5** | **0.01** | **5** | **16.7** | **32** | **46.4** | **0.005** | |
| The space is not inviting | 32.3 | 18 | 27.3 | 12 | 36.4 | 0.4 | 9 | 30.0 | 21 | 30.4 | >0.9 | |
| There is not enough space | 30.3 | **17** | **25.8** | **15** | **45.5** | **0.04** | 7 | 23.3 | 25 | 36.2 | 0.2 | |
| No access to water | 30.3 | 18 | 27.3 | 12 | 36.4 | 0.4 | 11 | 36.7 | 19 | 27.5 | 0.4 | |
| No private bathroom | 29.3 | 17 | 25.8 | 12 | 36.4 | 0.3 | 6 | 20.0 | 23 | 33.3 | 0.2 | |
| Do not know what to do out there | 26.3 | 15 | 22.7 | 11 | 33.3 | 0.3 | 7 | 23.3 | 19 | 27.5 | 0.7 | |
| Too hot/too cold (weather) | 26.3 | 15 | 22.7 | 11 | 33.3 | 0.3 | 11 | 36.7 | 15 | 21.7 | 0.1 | |
| Unmotivated | 26.3 | **12** | **18.2** | **14** | **42.4** | **0.01** | 7 | 23.3 | 19 | 27.5 | 0.7 | |
| No access to hygiene products | 23.2 | 15 | 22.7 | 8 | 24.2 | 0.9 | 6 | 20.0 | 17 | 24.6 | 0.6 | |
| No access to feminine products | 20.2 | 10 | 15.2 | 10 | 30.3 | 0.08 | 5 | 16.7 | 15 | 21.7 | 0.6 | |
| Detention officers invite us when they want to | 16.2 | 9 | 13.6 | 7 | 21.2 | 0.3 | 5 | 16.7 | 11 | 15.9 | 0.9 | |
| Detention officers do not come and get us | 13.1 | 9 | 13.6 | 4 | 12.1 | 0.8 | 2 | 6.7 | 11 | 15.9 | 0.2 | |
| Time of day is not good for me | 12.1 | 7 | 10.6 | 5 | 15.2 | 0.5 | 6 | 20.0 | 6 | 8.7 | 0.1 | |
|  |  |  |  |  |  |  |  |  |  |  |  | |
| **Benefits** |  |  |  |  |  |  |  |  |  |  |  | |
| Fresh air | 97.0 | 64 | 97.0 | 32 | 97.0 | >0.9 | 28 | 93.3 | 68 | 98.6 | 0.2 | |
| Vitamin D and Sunshine | 81.8 | 55 | 83.3 | 26 | 78.8 | 0.6 | 23 | 76.7 | 58 | 84.1 | 0.4 | |
| Good for my health | 71.7 | 47 | 71.2 | 24 | 72.7 | 0.9 | 22 | 73.3 | 49 | 71.0 | 0.8 | |
| Change in environment | 70.7 | 44 | 66.7 | 26 | 78.8 | 0.2 | **13** | **43.3** | **57** | **82.6** | **<0.001** | |
| Calmer | 68.7 | 47 | 71.2 | 21 | 63.6 | 0.4 | 21 | 70.0 | 47 | 68.1 | 0.9 | |
| Less anxious | 66.7 | 45 | 68.2 | 21 | 63.6 | 0.7 | **14** | **46.7** | **52** | **75.4** | **0.005** | |
| Less stressed or release stress | 64.7 | 42 | 63.6 | 22 | 66.7 | 0.8 | **15** | **50.0** | **49** | **71.0** | **0.04** | |
| Less depressed | 62.6 | 40 | 60.6 | 22 | 66.7 | 0.6 | **13** | **43.3** | **49** | **71.0** | **0.009** | |
| Improved attitude | 55.6 | 35 | 53.0 | 20 | 60.6 | 0.5 | 13 | 43.3 | 42 | 60.9 | 0.1 | |
| Hanging out | 51.5 | 33 | 50.0 | 18 | 54.6 | 0.7 | 11 | 36.7 | 40 | 58.0 | 0.05 | |
| Sleep better | 47.5 | **26** | **39.4** | **21** | **63.6** | **0.02** | 11 | 36.7 | 36 | 52.2 | 0.2 | |
| Burn excess energy | 46.5 | 32 | 48.5 | 14 | 42.4 | 0.6 | 12 | 40.0 | 34 | 49.3 | 0.4 | |
| Get along with other women | 41.4 | 25 | 37.9 | 16 | 48.5 | 0.3 | 9 | 30.0 | 32 | 46.4 | 0.1 | |
| Access to exercise equipment | 40.4 | 26 | 39.4 | 14 | 42.4 | 0.8 | 10 | 33.3 | 30 | 43.5 | 0.3 | |
| Less crowded | 40.4 | 24 | 36.4 | 15 | 45.5 | 0.4 | 11 | 36.7 | 28 | 40.6 | 0.7 | |
| Lose weight | 36.4 | 22 | 33.3 | 14 | 42.4 | 0.4 | 11 | 36.7 | 25 | 36.2 | >0.9 | |
| Less supervised | 15.2 | 11 | 16.7 | 4 | 12.1 | 0.6 | 3 | 10.0 | 12 | 17.4 | 0.3 | |
| Get along with detention officers | 15.2 | 9 | 13.6 | 6 | 18.2 | 0.6 | 4 | 13.3 | 11 | 15.9 | 0.7 | |
| Laxed rules | 13.1 | 9 | 13.6 | 4 | 12.1 | 0.8 | 2 | 6.7 | 11 | 15.9 | 0.2 | |
| ^a^ Physical health conditions included self-report of hypertension, high cholesterol, diabetes, and prediabetes  ^b^ Mental health conditions included anxiety, depression, bipolar disorder, schizophrenia, post-traumatic stress disorder, and attention deficit hyperactivity disorder  ^c^ Group differences in responses were assessed using chi square tests  Facilitators with less than 10 respondents: Do not want to be left out; Everyone else is doing it; Detention officers offer it on a schedule; I don’t feel safe staying in the dorm; Detention officers encourage it  Barriers with less than 10 respondents: I feel sick; I am too tired; No one else is going; I am not physically active; My friends do not want to go  Detention officers prevent me from going; I feel more supervised by detention officers; Someone else I do not like regularly attends rec-time; I do not feel safe at rec-time; Detention officers discourage me from going  There were no benefits with less than 10 respondents | | | | | | | | | | | | |
